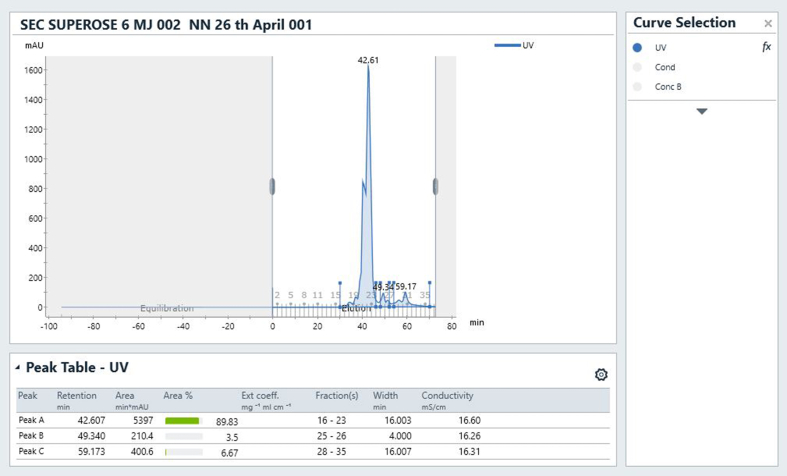# Non-compartmental toxicokinetic studies of the Nigerian *Naja nigricollis* venom

**DOI:** 10.1016/j.toxcx.2022.100122

**Published:** 2022-04-01

**Authors:** Auwal A. Bala, Sani Malami, Yusuf Abubakar Muhammad, Binta Kurfi, Ismaila Raji, Sanusi Muhammad Salisu, Mustapha Mohammed, George Oche Ambrose, Murtala Jibril, Jacob A. Galan, Elda E. Sanchez, Basheer A.Z. Chedi

**Affiliations:** a Department Pharmacology, College of Medicine and Health Sciences, Federal University Dutse, Jigawa, Nigeria; b Department of Pharmacology and Therapeutics, Bayero University Kano, Kano, Nigeria; c Department of Pharmacology, Bauchi State University Gadau, Bauchi, Nigeria; d Department of Biochemistry, Bayero University Kano, Kano, Nigeria; e Department of Pharmacology and Therapeutics, Faculty of Pharmaceutical Sciences, Ahmadu Bello University Zaria, Kaduna, Nigeria; f Department of Clinical Pharmacy and Pharmacy Practice, Faculty of Pharmaceutical Sciences, Ahmadu Bello University Zaria, Kaduna, Nigeria; g School of Pharmaceutical Sciences, Universiti Sains Malaysia, Penang, Pulau Pinang, Malaysia; h Centre for Malaria and Other Tropical Diseases, University of Ilorin Teaching Hospital, Ilorin, Kwara, Nigeria; i National Natural Toxin Research Center (NNTRC), Texas A&M University-Kingsville, Kingsville, USA; j Venom-Antivenom Research Project (VASP) and Nigeria- Snakebite Research and Intervention Centre (N- SRIC), Nigeria

**Keywords:** Snake venom, Antisnake venom, Toxicokinetics, ELISA, *Naja nigricollis*, Pharmacometrics

## Abstract

Snakebite envenoming (SBE) is a neglected public health problem, especially in Asia, Latin America and Africa. There is inadequate knowledge of venom toxicokinetics especially from African snakes. To mimic a likely scenario of a snakebite envenoming, we used an enzyme-linked immunosorbent assay (ELISA) approach to study the toxicokinetic parameters in rabbits, following a single intramuscular (IM) administration of Northern Nigeria *Naja nigricollis* venom. We used a developed and validated non-compartmental approach in the R package PK to determine the toxicokinetic parameters of the venom and subsequently used pharmacometrics modelling to predict the movement of the toxin within biological systems. We found that *N. nigricollis* venom contained sixteen venom protein families following a mass spectrometric analysis of the whole venom. Most of these proteins belong to the three-finger toxins family (3FTx) and venom phospholipase A_2_ (PLA_2_) with molecular weight ranging from 3 to 16 kDa. Other venom protein families were in small proportions with higher molecular weights. The *N. nigricollis* venom was rapidly absorbed at 0.5 h, increased after 1 h and continued to decrease until the 16th hour (T*max*), where maximum concentration (C*max*) was observed. This was followed by a decrease in concentration at the 32nd hour. The venom of *N. nigricollis* was found to have high volume of distribution (1250 ± 245 mL) and low clearance (29.0 ± 2.5 mL/h) with an elimination half-life of 29 h. The area under the curve (AUC) showed that the venom remaining in the plasma over 32 h was 0.0392 ± 0.0025 mg h.L^−1^, and the mean residence time was 43.17 ± 8.04 h. The pharmacometrics simulation suggests that the venom toxins were instantly and rapidly absorbed into the extravascular compartment and slowly moved into the central compartment. Our study demonstrates that Nigerian *N. nigricollis* venom contains low molecular weight toxins that are well absorbed into the blood and deep tissues. The venom could be detected in rabbit blood 48 h after intramuscular envenoming.

## Highlights

• Toxicokinetics of *Naja nigricollis* venom were determined in rabbits.
• A non-compartmental pharmacokinetics approach was used.
• Venomics analysis of *Naja nigricollis* major toxins families.
• Our study suggests distribution of toxins into deep tissues.

## Graphical abstract

**Figure:**
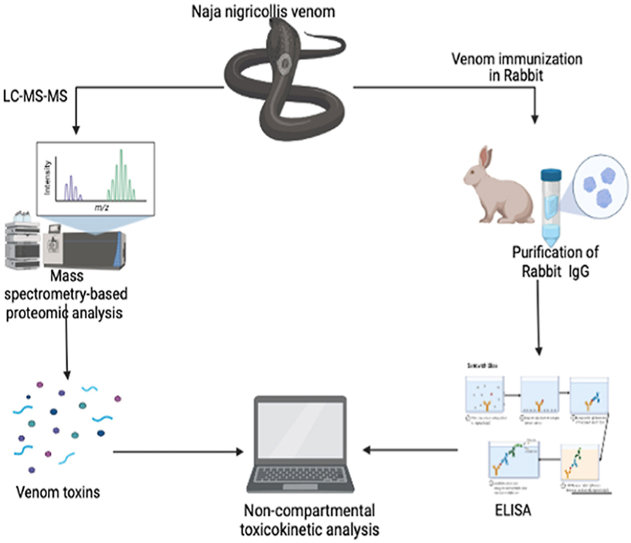

## 1 Introduction

Snakebite envenoming (SBE) is a neglected public health problem, especially in Asia, Latin America and Africa (WHO, 2017) and is on the rise in Nigeria and other West African countries mainly due to paucity of anti-snake venom, poor treatment knowledge, and inadequate treatment centres (Michael et al., 2018; Bala et al., 2021; Gutiérrez et al., 2021). The SBE affects poor communities in Asia and Africa, leading to compounded disadvantages with negative impacts on the economy and the healthcare sectors of the countries in the regions (Habib and Brown, 2018). A recent study estimated the burden of SBE at 1.03 million disability-adjusted life years per annum in sub-Saharan African, out of which Nigeria has the highest-burden with 43% of the total burden in West Africa (Halilu et al., 2019).

Indigenous to Africa, the cobras, belonging to the Elapidae family, represent the most medically essential snakes (WHO, 2017). Generally, spitting cobras have active venom that causes ulceration and necrosis around the bite site, with systemic neurotoxic effects. If there is significant tissue damage in the bitten limb, secondary fluid shifts may result in shock, and its sequelae and secondary infection and long-term scarring are common (Warrell, 1999; Chippaux et al., 2016). Hospital records have shown that the black-necked spitting cobra (*Naja nigricollis*) is the most urbanized and medically important elapid snake in northern Nigeria (Habib, 2003; Yusuf et al., 2015).

Despite the availability of institutional SBE treatment protocols in some medical centres, many centres do not have well-optimized SBE treatment protocols due in part to inadequate knowledge of the toxicokinetics of medically important snake venoms, especially in some affected communities of sub-Saharan Africa (Chippaux, 1998; Yap et al., 2014). To fully understand the pathophysiology and improve the treatment protocol of cobra envenoming, it is imperative to understand the toxicokinetics of the medically important snake venoms (Yap et al., 2013; Sanhajariya et al., 2018). There is an urgent need to improve the determination of antisnake venom dosing and timing of administration because, in many cases, the dose administered is determined based on clinical symptoms, laboratory results, and sometimes animal studies (Maduwage et al., 2016). The study of the time course of venom in biological systems will provide important information about the time course of the patients envenoming (Sanhajariya et al., 2018).

Theakston et al. (1977) first developed the use of enzyme-linked immunosorbent assay.

(ELISA) in detecting venom in biological samples, and it was found to have good sensitivity and specificity (Selvanayagam and Gopalakrishnakone (1999); Theakston, 1983). Recently, the sandwich ELISA approach represented a good approximation of the true picture of the toxicokinetics of snake venom using primary antibodies that binds to major toxins (Tan et al., 1993; Yap et al., 2013). Toxicokinetic analyses have been reported using the ELISA technique for some Asian cobras, including *Naja sumatrana, Naja suputatrix, Pseudechis australis, Naja atra* (Tseng et al., 1968; Yap et al., 2013; Hart et al., 2014). Another study in Asia used radioimmunoassay and reported a three compartmental toxicokinetic analysis for some cobra venoms, including *Naja melanoleuca, Naja nivea, Naja nigricollis and Naja haje* (Ismail et al., 1996)**.** Thus, our study employed a non-compartmental analysis (NCA) because it is faster, cost efficient and most importantly requires fewer or no assumptions compared with compartmental-based approaches as previously described (Gabrielsson and Weiner 2012; Jaki and Wolfsegger 2012). To mimic a likely scenario of a snakebite envenoming, we used an ELISA approach to study the toxicokinetics of *N. nigricollis* venom captured from Northern Nigeria following a single intramuscular administration in rabbits. We used a developed and validated non-compartmental approach in the R package PK to determine the toxicokinetic parameters of the venom and subsequently used pharmacometrics modelling to predict the movement of the toxins within the biological systems.

## 2 Materials and methods

### 2.1 Ethics statement

The Ethics Committee of the College of Health Sciences, Bayero University Kano approved the study (BUK/CHS/HREC/VII/66). All animals were treated according to the WHO's ethical code for animal experimentation (Howard-Jones 1985).

### 2.2 Animals

Swiss albino mice weighing 18–20 g and New Zealand rabbits weighing 2.5–2.7 kg were obtained from Bayero University Kano and maintained according to Bayero University guidelines for the care and use of laboratory animals at the Animal House Facility, Department of Pharmacology and Therapeutics, Bayero University Kano, Nigeria.

### 2.3 Snake specie

Five *N. nigricollis* were captured from the wild in northern Nigeria. They were identified and housed at the Faculty of Veterinary Medicine Herpeterium, Ahmadu Bello University, Zaria, Kaduna, Nigeria.

### 2.4 Venom collection

Venoms from the five captured *N. nigricollis* were manually pooled at Veterinary Medicine Herpetarium, Ahmadu Bello University Zaria, Kaduna, Nigeria, using the method described by Markfarlane (1967).

### 2.5 Proteomic analysis of *N. nigricollis* venom

#### 2.5.1 Mass spectrometric analysis of *N. nigricollis* whole venom

##### 3.5.1.1 Sample preparation

One microgram of snake venom was denatured in 0.1% RapiGest (Waters, Milford, MA, USA) and reduced with 5 mM dithiothreitol for 30 min at 50 °C. Proteins were alkylated in 15 mM iodoacetamide for 1 h in the dark at room temperature and then digested with proteomics-grade trypsin at a 1:100 ratio overnight at 37 °C. The pH was adjusted below 3, and the sample was incubated for 45 min at 37 °C. The sample was centrifuged at 16,000×*g* to remove RapiGest. The supernatant was collected, and peptides were dissolved in 5 μL of 0.25% formic acid (FA) with 3% CAN (Willard et al., 2021*).*

##### 3.5.1.2 Liquid chromatography-tandem mass spectrometry (LC–MS/MS)

The LC-MS-MS analysis was conducted as described by Willard et al. (2021)*.* Samples were dried completely in a vacuum centrifuge and stored at −80 °C. One microgram of each dried peptide sample was dissolved in 10.5 μL of 0.05% trifluoroacetic acid with 3% (vol/vol) acetonitrile. In total, 10 μL of each sample was injected into an Ultimate 3000 nano UHPLC system (Thermo Fisher Scientific, Vantaa, Finland). Peptides were captured on a 2 cm Acclaim PepMap trap column and separated on a heated 50 cm column packed with ReproSil Saphir 1.8 μm C18 beads (Dr Maisch GmbH, Ammerbuch, Germany). The mobile phase buffer consisted of 0.1% formic acid in ultrapure water (buffer A) with an eluting buffer of 0.1% formic acid in 80% (vol/vol) acetonitrile (buffer B) ran with a linear 60 min gradient of 6–30% buffer B at a flow rate of 300 nL/min. The UHPLC was coupled with a Q Exactive HF-X mass spectrometer (Thermo Fisher Scientific). The mass spectrometer was operated in the data-dependent mode, in which a full-scan MS (from m/z 375 to 1500 with the resolution of 60,000) was followed by MS/MS of the 15 most intense ions (30,000 resolution; normalized collision energy—28%; automatic gain control target (AGC)—2E4: maximum injection time—200 ms; 60 s exclusion). The raw files were searched directly against the Naja/Echis (384/74) in UniProt with no redundant entries, using Byonic (Protein Metrics) and SEQUEST search engines loaded into Proteome Discoverer 2.3 software (Thermo Fisher Scientific). MS1 precursor mass tolerance was set at 10 ppm, and MS2 tolerance was set at 20 ppm. Search criteria included a static carbamidomethylation of cysteines (+57.0214 Da) and variable modifications of oxidation (+15.9949 Da) on methionine residues and acetylation (+42.011 Da) at the N-terminus of proteins. A search was performed with full trypsin/P digestion and allowed a maximum of two missed cleavages on the peptides analyzed from the sequence database. The false-discovery rates of proteins and peptides were set at 0.01. All protein and peptide identifications were grouped, and any redundant entries were removed. Unique peptides and unique master proteins were reported.

#### 2.5.2 Separation of *N. nigricollis* major toxins

Fresh, lyophilized crude venom of *N. nigricollis* was dissolved in 1 mL Phosphate-buffered saline (PBS) to a final concentration of 10 mg/mL. The venom's size exclusion chromatography was carried out using fast protein liquid chromatography (FPLC) with ÄKTA pure 25^MT^. The column (24 mL Superose 6 column 10/300 GL {GE Healthcare} and a 0.5 mL loop was slowly equilibrated at 0.2 mL/min to remove the storage buffer (20% ethanol) with MilliQ water (2 column volume) then at 0.5 mL/min with PBS (2 column volume). The loop was also equilibrated with MilliQ water followed by PBS. Pump washes were carried out using MilliQ water and PBS. During size-exclusion chromatography, the sample injection volume was 250 μL (50% of the loop volume), and the elution buffer was PBS pH 7.4 at a flow rate of 0.5 mL/min. Fractions were collected into tubes and were stored at −20 °C in aliquots before molecular weight determination using 15% Sodium Dodecyl Sulfate Polyacrylamide Gel Electrophoresis (SDS-PAGE).

##### 3.5.2.1 Molecular weight determination of *N. nigricollis* venom toxins

The molecular weight of the separated fractions of *N. nigricollis* venom and the purified anti- *N. nigricollis* IgG were checked using sodium dodecyl sulfate polyacrylamide gel electrophoresis (SDS-PAGE) as described by Laemmli (1970). A broad range SDS-PAGE standard (250–10 kDa; Bio-Rad™) and stained with Coomassie Brilliant Blue G 250 for 4 h. Destaining was conducted with a destaining solution as described by the manufacturer (Solarbio ™).

### 2.6 Toxicokinetics studies of *N. nigricollis* venom

#### 2.6.1 Production and purification of polyclonal IgG against *Naja nigricollis* venom

Three New Zealand rabbits were immunized with the *N. nigricollis* venom as described by Yap et al. (2013). Fifty micrograms (50 μg) of the venom in 0.2 mL of PBS was mixed with an equal volume of Complete Freund's adjuvant (Sigma), and the three rabbits were immunized with the whole volume by intramuscular (IM) injection through the quadriceps muscles. The second and third immunizations were performed on days 14 and 28 with Freund's incomplete adjuvant (Sigma), and finally on day 35. After the last immunization, 20 mL of blood was collected from the rabbit with the highest IgG titer through the jugular vein. The collected blood was allowed to clot by leaving it undisturbed at room temperature for about 15 min and then centrifuged at 3000×*g* for 5 min. The resulting serum was immediately transferred into a clean container tube using a Pasteur pipette and maintained at −20 °C for further use.

##### 3.6.1.1 Precipitation of rabbit polyclonal IgG (ammonium sulfate)

The collected serum was precipitated using a 40% ammonium sulfate and loaded onto an affinity chromatography column as described by Mariam et al. (2015). The IgG containing serum was transferred to a beaker and stirred. Saturated ammonium sulfate was slowly added to stir the sample to bring the final concentration to 50% saturation. The sample was kept at 4 °C overnight, after which it was centrifuged at 3000 g for 30 min. The supernatant was carefully removed and discarded. The residue (pellet) was suspended in 50% of the starting volume in 1X PBS.

##### 3.6.1.2 Dialysis and affinity chromatography

The precipitated antibody solution was transferred into an 8000–14,000 wt dialysis tube (Solarbio ™) and dialyzed against three changes of tris - phosphate buffer (pH 8.1). The dialyzed anti - *N. nigricollis* venom IgG was further purified using affinity chromatography as described by Hudson and Hay (1980). Two millilitres (2 mL) of protein A-Sepharose was packed into a small chromatography column (Solarbio ™), then 5 mL serum was diluted with an equal volume of PBS and filtered through the column at a flow rate of 6 mL/h for 30 min. The column (unbound protein) was then washed with PBS and the bound IgG was eluted with glycine–HCl buffer (pH 2.8). The concentration was checked using a spectrophotometer and the purity was checked with SDS PAGE under reducing condition.

### 2.7 Determination of the antigenic reactivity of the purified IgG used in ELISA

The affinity of the purified IgG against *N. nigricollis* crude venom and the separated fractions were observed using indirect ELISA. The ELISA microplate was coated with sodium carbonate/bicarbonate buffer pH 9.6 (solarbio^MT^) in six serial dilutions of 1000 ng/mL of crude venom and each fraction, and were incubated overnight at 4 °C. The plate was blocked with 5% BSA (Solarbio™) and washed with PBS–Tween 20. Anti-*N. nigricollis* IgG (dilution of 1:500) was added and allowed to incubate at room temperature for 1 h. It was followed by incubation with 100 μL goat anti-rabbit IgG horseradish peroxidase conjugate and 100 μL of TMB substrate for 1 h. The reaction was then terminated by adding 50 μL sulfuric acid (12.5%). The absorbance at 492 nm was determined using an EnSight multimode plate reader (PerkinElmer, USA).

### 2.8 Determination of serum venom (antigen) using sandwich ELISA

Three rabbits were injected with 0.5 mg/kg of venom intramuscularly through the quadriceps muscles and blood samples were collected through the marginal ear vein at 0.5, 1, 2, 4, 8, 16 and 32 h. The serum antigen concentrations at specific times were measured using sandwich ELISA as described by Tan et al. (1993). ELISA microplates were coated overnight at 4 °C with 100 μL of the anti- *N. nigricollis* IgG (4 mg/mL) using sodium carbonate/bicarbonate buffer pH 9.6 (Solarbio^MT^). The plates were blocked with 5% BSA (Solarbio™) and subsequently incubated with 100 μL of diluted serum samples (1:50) collected at different time intervals. The plates were washed with PBS–Tween and subsequently anti-*N. nigricollis* IgG (dilution of 1:500) was added and allowed to incubate at room temperature for 1 h. It was followed by incubation with 100 μL goat anti-rabbit IgG horseradish peroxidase conjugate (1:1000) for 2 h, and 100 μL of 3,3′5,5′- Tetramethylbenzidine (TMB) substrate was added. The reaction was terminated 1 h later by adding 50 μL sulfuric acid (12.5%), and the absorbance was determined at 492 nm using a microplate reader. A standard curve was constructed using serial dilutions of venom in pre-envenomed sera (1000, 500, 250, 125, 62.5, 31.25, 15.63, and 7.81 ng/mL).

### 2.9 Pharmacokinetic and pharmacometrics analysis

Non-compartmental pharmacokinetics (PK) analysis was conducted using the R Core Team (2020), and data were presented as mean ± standard error of the mean (SEM). PK parameters of interest were estimated as described by Jaki and Wolfsegger (2011). The mrgsolve package in R was also used to evaluate the venom movement following a simulation approach. The Vd and CL estimated from the non-compartmental approach were used to simulate the distribution in the First extravascular compartment (EV1), Central compartment (CENT, Mass) and Plasma concentration (CP, mass/volume) as described by Elmokadem et al. (2019).

## 4 Results

### 4.1 Median lethal dose (LD_50_)

**Fig. 1:**
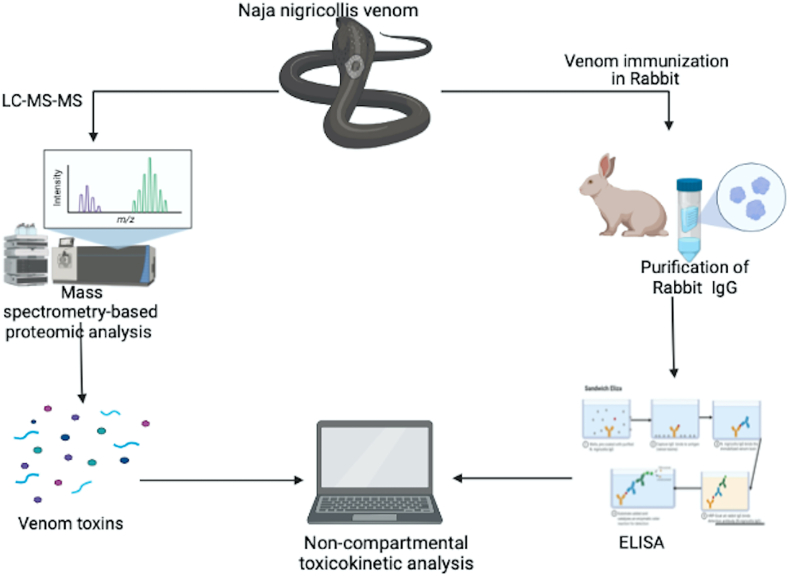
Graphical illustration of the study.

The median lethal dose (LD_50_) of *N. nigricollis* venom estimated intraperitoneolly (IP) using probit analysis was found to be 1.0 mg/kg in mice (see Fig. 1).

### 4.2 Development of polyclonal IgG against *N. nigricollis* venom

**Fig. 2:**
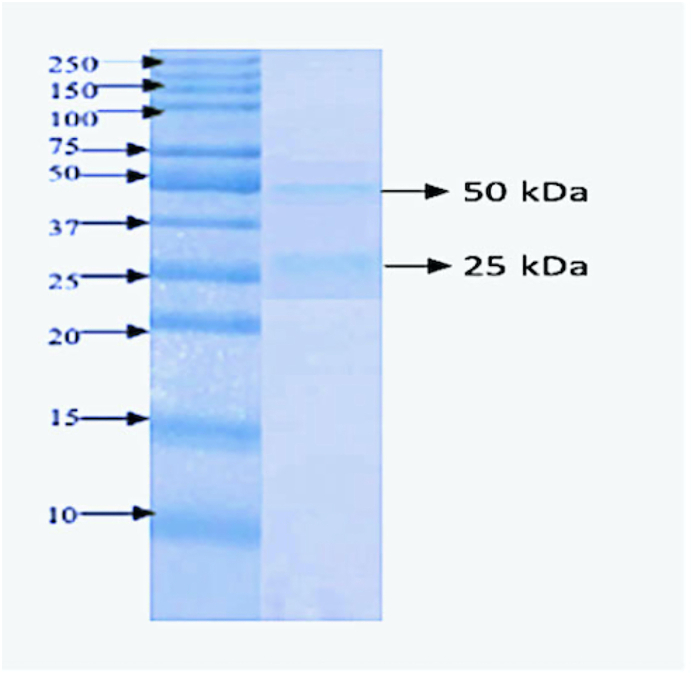
SDS PAGE of purified rabbit IgG.

The concentration of the developed and purified polyclonal IgG against *N. nigricollis* venom was 13 mg/mL and the purity was demonstrated using SDS PAGE as shown on Fig. 2.

### 4.3 Proteome of *N. nigricollis* venom

**Fig. 3:**
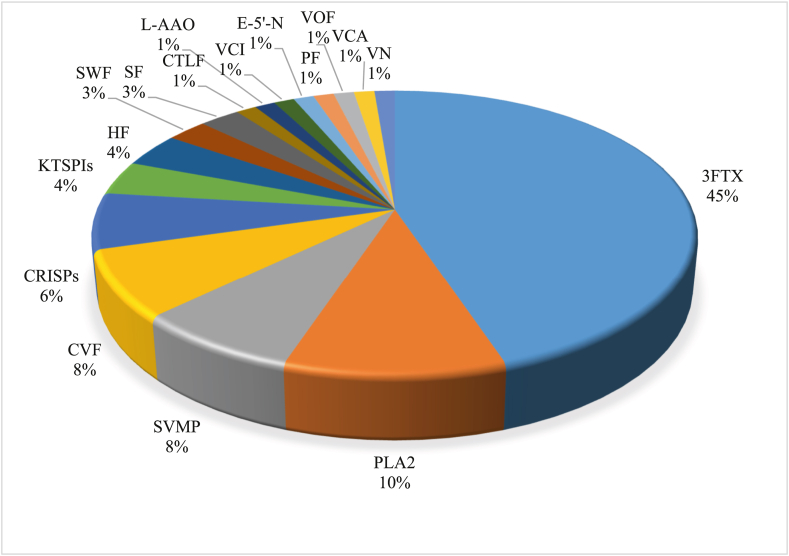
Percentage of venom families found in *Naja nigricollis* venom. ***3FTX*** = *Three Finger Toxins,****PLA***_***2***_ = *Phospholipase A*_*2*_*,****SVMP*** = *Snake Venom Metalloproteinases****CVF*** = *Cobra Venom Factor,****CRISPs*** = *Cysteine-Rich Secretory Proteins,****KTSPIs*** = *Kunitz-Type Serine Proteise Inhibitor,****HF*** = *Hydrolase Family,****SWF*** = *Snake Waprin Family,****SF*** = *Serine Family,****CTLF*** = *C-Type Lectin Like Family,****L-AAO*** = *L-amino acid oxidase,****VC*** = *Venom Cystatin,****PF*** = *Phosphodiesterase Family,****VOF*** = *Venom Ohanin Family,****VC*** = *Venom Cathelcidin*

Proteomic analysis of *N. nigricollis* venom revealed sixteen (16) venom protein families with a total number of seventy-seven (77) proteins. Most of these proteins belong to the three-finger toxins family (3 TFx) and Venom phospholipase A2 (PLA_2_) with molecular weight ranging from 3 to 16 kDa. Other families are in small proportions with higher molecular weights, as shown in Fig. 3.

### 4.4 Separation of *N. nigricollis* toxins for indirect ELISA

**Fig. 4:**
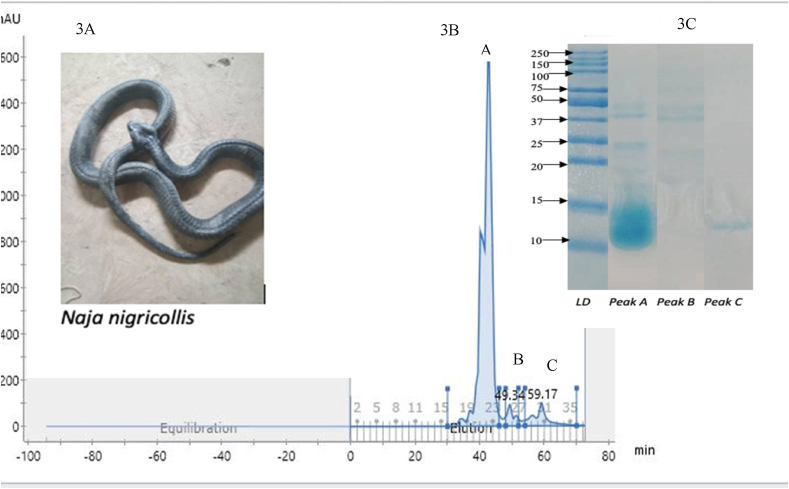
Size exclusion fractionation of *N. nigricollis* venom and SDS PAGE analysis of its fractions. 3 A) *Naja nigricollis* in image. 3 B) size exclusion chromatogram 3C) SDS-PAGE of the three fractions.

Three peaks (fractions) were collected from the Fast Protein Lipid Chromatography. The SDS PAGE analysis of these peaks revealed proteins with lower molecular weights of 7–15 kDa and a few with higher molecular weights proteins 28–37 kDa, as shown in (Fig. 4). The molecular weights of proteins in Peak A were found to be similar to the 3FTx, PLA_2_ and CRISP, Peak B were within the serine & cathelcidin family as revealed in the mass spectrometric analysis, while Peak C contains 3FTx and PLA_2._

### 4.5 Indirect ELISA of *Naja nigricollis* whole venom and its fractions

**Fig. 5:**
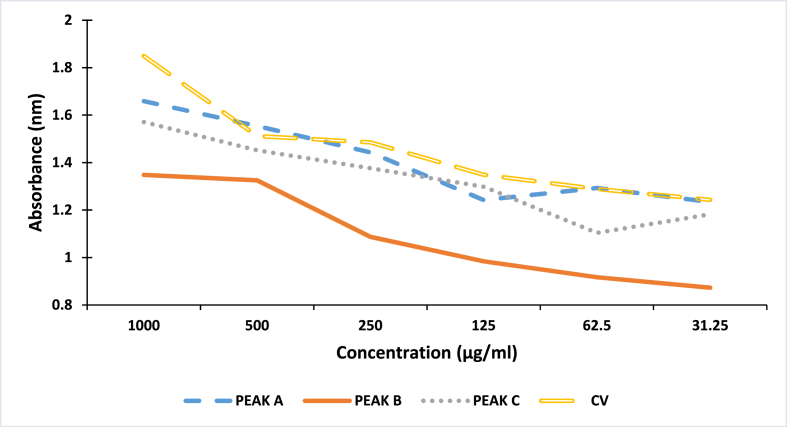
Affinity of purified IgG against *Naja nigricollis* venom and separated toxins.*CV* = *Crude venom. A* = *Fraction A, B* = *Fraction B, C* = *Fraction C*.

Indirect ELISA demonstrated the affinity of the Purified *N. nigricollis* IgG against venom and the separated fractions (Fig. 5). We also found higher affinity of the IgG against the crude venom followed by fraction A, fraction C and fraction B.

### 4.6 Sandwich ELISA for quantification of venom serum concentration

Venom serum concentration was detected 30 min after venom administration (1364 ± 167 ng/mL), and it increases until it reaches the maximum plasma concentration (1813 ± 175 ng/mL) at the 16th hour (Table 1).

**Table 1:** Serum venom concentration of *N. nigricollis* using sandwich ELISA.

**Time (hours)**	0.5	1	2	4	8	16	32
**Conc (ng/ml)**	1364 ± 167	1448 ± 180	1253 ± 230	1134 ± 75	1105 ± 215	1813 ± 175	614 ± 22

Time = time of measurement post administration, Con. = concentration of serum venom, ng/ml = nanogram per millimeter ± standard error of mean, rabbit weight (n = 3), 2.2–2.3 kg, Dose of venom injected = 0.5 mg/kg (intramuscular).

### 4.7 Toxicokinetic parameters of *Naja nigricollis* venom

The venom AUC_t32_ and (AUC∞) were determined to be 0.0392 ± 0.0025 and 0.0691 ± 0.0059 mg/h/L^−1^, respectively. The elimination half-life (29.92 ± 5.57 h), clearance (28.95 ± 2.45 mL/h), volume of distribution (1249.64 ± 245.33 mL), and mean residence (43.17 ± 8.04 h) following the non-compartmental pharmacokinetic analysis as shown in Table 2.

**Table 2:** Toxicokinetic parameters of *Naja nigricollis* venom.

Parameter	Value
AUC_t32_ (mg/hr/L^−1^)	0.0392 ± 0.0025
AUC∞ (mg/hr/L^−1^)	0.0691 ± 0.0059
Mean residence time (hr)	43.17 ± 8.04
Elimination Half-life (hr)	29.92 ± 5.57
Clearance (mL/hr)	28.95 ± 2.45
Volume of distribution (mL)	1249.64 ± 245.33
C*max* (ng/mL)	1813 ± 175
T*max* (hr)	16
Elimination rate constant (K10) (hr^−1^)	0.0232

### 4.8 Pharmacometrics simulation

**Fig. 6:**
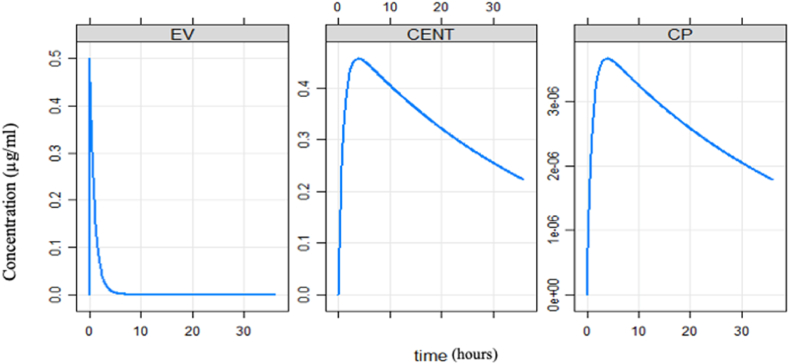
Simulated redistribution of toxin between compartments. EV = extravascular compartments, CENT = Central compartments, CP = Plasma concentration.

The pharmacometrics simulation shows that the venom was instantaneously distributed into the extravascular compartment compared to the central compartment (Fig. 6).

## 5 Discussion

The LC-MS-MS analysis of the crude venom revealed 16 protein families, with the majority belonging to 3FTx (45%), PLA2 (10%) followed by SVMP (8%), CVF (8%) and CRISPs (6%). The venom protein families in our study are similar to what has been reported by previous studies (Petras et al., 2011; Tasoulis and Isbister, 2017; Adamude et al., 2021). Clinically, CRISPs & PLA_2_ have been reported to penetrate into the deep tissues and induce necrosis while 3FTx & PLA_2_ have been implicated in neurotoxicity and cytotoxicity (Linda and David 1975; AdinaChaim-Matyas and Ovadia, 1987; Kuzun et al., 1994; Conlon et al., 2020). To confirm that our purified primary anti- *N. nigricollis* IgG binds to the important toxins in the crude venom, we fractionated some of the major and clinically implicated toxins (3FTx, PLA_2_ & CRISPs) using size exclusion chromatography and confirmed their molecular weights using SDS PAGE. The indirect ELISA conducted against these fractionated toxins has made it possible to monitor the serum venom concentration post administration of the venom. The developed and purified IgG shows binding affinity to the fractions in a similar way to the crude venom.

### 5.1 Toxicokinetics

In clinical settings, the victim's venom blood level is rarely studied. Still, these data have been reported in studies involving laboratory animals using immunoassays such as ELISA and radioimmunoassay (Theakston et al., 1977; Theakston and Laing, 2014; Sanhajariya et al., 2020). The compartmental approach represent the body as a system of compartments, even though these compartments usually have no absolute physiologic or anatomic reality, this approach make certain assumptions such as; the body is made up of one, two or multiple compartments, they also assume that the rate of transfer between compartments and the rate of drug elimination from compartments follow first-order or linear kinetics (Gabrielsson and Weiner, 2012; Qusai et al., 2016). In our study, we employed a non-compartmental analysis (NCA) because it is faster, cost efficient and most importantly requires fewer or no assumptions compared with compartmental-based approaches as previously described (Gabrielsson and Weiner 2012; Jaki and Wolfsegger 2012). Based on our study, the volume of distribution (Vd) of the crude venom was found to be higher (1250 ± 245 mL) compared with the average blood of rabbit 57–65 mL/kg. It therefore, suggests that the majority of the toxins can penetrate deep into the extravascular tissues, as reported in some cobra venom from Asia (Tseng et al., 1968; Guo et al., 1993; Ismail et al., 1996; Yap et al., 2014). This could be as a result of the low molecular weight (7–37 kDa) of the toxins (Tasoulis and Isbister, 2017; Conlon et al., 2020; Adamude et al., 2021). The knowledge of venom Vd could play a vital role in the timing and selection of appropriate antisnake venom (ASV), because of difference in formulations of ASV which can either contain fragmented [F (ab')2], Fab’ or intact IgG (Ismail et al., 1998; WHO 2017). The fragmented IgGs have smaller molecular weights and larger Vds than the intact IgGs, implying that *N. nigricollis* venom might be better neutralized in the deep tissues with ASV that contains IgG fragments (F (ab')2s or Fab's). The terminal half-life (t_1/2_) of the *N. nigricollis* venom (29.9 h) was found to be higher than those reported from Asian Cobras (12–22 h) even though those studies used either two or multiple compartmental approaches in determining the toxicokinetic parameters (Tseng et al., 1968; Guo et al., 1993; Yap et al., 2014). The of high Vd, t_1/2_ and low clearance (29 ± 246 mL/h) is an indication that the slow absorption and deep tissue distribution might be the cause of the slow elimination of cobra venom. Similar elimination t_1/2_ in both intravenous and intermuscular injection were reported in Asian cobras (Yap et al., 2014).

### 5.2 Pharmacometric simulation

Previous studies showed that snake venom toxins distribute extensively to the peripheral or extravascular tissues, which seems to be a general phenomenon for venom toxins (Barral-Netto and von Sohsten, 1991; Guo et al., 1993; Audebert et al., 1994). The simulation results also indicated that *N. nigricollis* venom does not follow a single compartmental model of kinetics, especially when we look at serum venom concentration profile, the rapid absorption within 0.5 h (1364 ± 167 ng/mL) which increased after 30 min (1448 ± 180 ng/mL) and subsequently started reducing until the 16th hour where we observed the sharp increase to the maximun concentarrion (1813 ± 175 ng/mL), this was followed by a decrease in concentration (614 ± 22 ng/mL) up to the 32nd hour. The increase in concentration at the 16th hour may explained by the redistribution of the low molecular weight toxins back into the blood circulation from the extravascular system. This redistribution of venom toxins may be related to the rebound phenomenon that sometimes occurs during ASV therapy (Gutierrez et al., 2003; Chippaux 2006). Experimental studies in rabbits have indicated that ASV therapy can cause a redistribution of venom from the extravascular to the vascular compartment. A good antisnake venom will be immediately sequestered by venom antibodies (Choumet et al., 1996; Rivière et al., 1997).

### 5.3 Limitation

We only used a single intramuscular (IM) administration of venom, which limited us in determining distribution half-life and actual bioavailability of the venom after administration.

### 5.4 Conclusion

We found that Nigerian *N. nigricollis* venom contains low molecular weight toxins that are well absorbed into rabbit blood and deep tissues and were detected 48 h after intramuscular envenoming.

## Statement of authorship

We declared that this work was conducted by the authors named in this article, and all liabilities relating to the content of this article were borne by them.

**Auwal A. Bala** conceived the original idea, developed the theory and co-developed the methods. **Sani Malami, Murtala Jibril, Binta Kurfi, Jacob A. Galan, Elda E. Sanchez and Basheer A.Z. Chedi,** developed the study methods and co-supervised the work. **Auwal A. Bala and Yusuf Abubakar,** performed laboratory work and co-wrote the manuscript**. George Oche Ambrose and Mustapha Mohammed** designed and conducted the formal statistical analysis**. Ismaila Raji and Sunusi Muhammad** conducted literature review and co-wrote the introduction. **Jacob A. Galan** performed the proteomic analysis and reviewd the manuscript. **Elda E. Sanchez** edited and critically reviewed the manuscript for intellectual content, **Basheer AZ. Chedi.,** gave the supervisory approval and finally revised the manuscript for intellectual content.

## Funding

This research was funded by a grant from the 10.13039/100000002NIH/10.13039/100016958ORIP, Viper Resource Grant #P40OD01960-18, Elda E. Sánchez, 10.13039/100000002NIH/SCGM136606-02, Jacob Galan and the Robert A.Welch Foundation, grant # AC-0006 (10.13039/100010859TAMUK—Department of Chemistry).

## Statement of authorship

We declared that this work was conducted by the authors named in this article. **Auwal A. Bala** and **Basheer A.Z. Chedi** conceived the original idea, developed the theory and co-developed the methods. **Sani Malami, Murtala Jibril, Binta Kurfi, Jacob A. Galan, Elda E. Sanchez and Basheer A.Z. Chedi** developed the study methods and co-supervised the work. **Auwal A. Bala and Yusuf Abubakar** performed laboratory work and co-wrote the manuscript**. George Oche Ambrose and Mustapha Mohammed** designed and conducted a formal statistical analysis**. Ismaila Raji and Sunusi Muhammad** conducted the literature review and co-wrote the introduction. **Jacob A. Galan** performed the proteomic analysis and reviewed the manuscript. **Elda E. Sanchez** edited and critically reviewed the manuscript for intellectual content, **Basheer AZ. Chedi** gave the supervisory approval and finally revised the manuscript for intellectual content.

## Ethical statement

The study was approved by the Ethics Committee of the College of Health Sciences, Bayero University Kano (BUK/CHS/HREC/VII/66). All animals were treated according to the WHO's ethical code for animal experimentation (Howard-Jones 1985).

## Declaration of competing interest

The authors declare no competing interest.

## Figures and Tables

**figs1:**